# Reply to Kaldalu et al., “Reanalysis of Proteomics Results Fails To Detect MazF-Mediated Stress Proteins”

**DOI:** 10.1128/mBio.01068-19

**Published:** 2019-06-11

**Authors:** Akanksha Nigam, Tamar Ziv, Adi Oron-Gottesman, Hanna Engelberg-Kulka

**Affiliations:** a Department of Microbiology and Molecular Genetics, IMRIC, The Hebrew University Hadassah Medical School, Jerusalem, Israel; b Smoler Proteomics Center, Faculty of Biology, Technion, Haifa, Israel; Harvard University

**Keywords:** toxin-antitoxin, bacterial stress response, proteomics

## REPLY

We thank Niilo Kaldalu and his coworkers in the lab of Tanel Tenson for taking the time to offer comments regarding our paper “Stress-Induced MazF-Mediated Proteins in Escherichia coli” (1), but we disagree with the points they raised, and we respond to all their questions as follows.

We compared the wild-type (WT) strain with its Δ*mazEF* derivative only in the presence of NA (nalidixic acid) and not in the absence of NA, because as we have shown previously (2, 3), MazF is induced only in the presence of stress, for example, NA. Also, MazF induction by stress was well established by us by the use of a GFP-STM (green fluorescent protein-stress translation machinery) reporter (2). Therefore, we did not do a control for NA again. According to the STM hypothesis that we discovered (4), MazF cleaves off the anti-Shine-Dalgarno (SD) sequence from the 3′ end of the 16 rRNA in the mature 30S ribosome, creating defective ribosomes. This was done by cleaving an ACA site located 43 nucleotides from the 3′ side of the mature 16S rRNA. In addition, we initially showed (4) that MazF also cleaves selected mRNAs within 5′ leaderless sequence to produce a pool of leaderless sequences (A↓CAUG) when the ACA is located immediately upstream of the AUG initiator called leaderless mRNAs (4). Subsequently, it was shown by Sauret et al. (5) that the stress-induced MazF-mediated defective ribosome is translating not only leaderless mRNAs (A↓CAUG) but also all selected mRNAs carrying an ACA site up to 100 nucleotides upstream of the AUG initiator that are cleaved by MazF. This was confirmed in our criticized proteomic study (1). In this paper (1), we showed that the mRNAs of most of the proteins obtained (36 out of 42) carry an ACA site up to 100 nucleotides upstream of the AUG initiator. Thus, our criticized proteomic paper confirms our previous papers on the stress-induced MazF-mediated translation machinery (4, 5).

Previous investigators failed to detect the deficient 30S ribosomes of STM, because they applied MazF to purified 16S rRNA (containing no ribosomal proteins). Purified 16S rRNA produces internal base pairing that is resistant to MazF cleavage. We applied MazF to whole 30S ribosomes (containing 16S rRNA and 30S ribosomal proteins) (4). Such whole ribosomes are cleaved by MazF, which produces the deficient ribosomes by MazF cleavage (4).

Kaldalu and coworkers also raised questions about our proteomic experiment and its statistical analysis. In response to Kaldalu and coworkers, we want to answer that for our proteomic results, the data were presented in a simplified way, as this journal addresses a general community that might not be familiar with proteomic data. A short time point was used to try and find the direct effectors.

As most of the proteins are not synthesized by the STM system, most of the proteins will not have a ratio. However, it is not correct to look only at the ratio data, thus eliminating some of the most interesting proteins that were translated in only one of the group. Thus, we also looked at the intensity data, taking into account proteins that had intensity quantification but not ratios. A total of 317 proteins were newly synthesized, not 125 as used in the reanalysis of Kaldalu and coworkers.

We are enclosing the *t* test analysis (see Data Set S1 in the supplemental material). The missing heavy/light (H/L) ratios of proteins that were quantified only in the light isotopes were replaced with 0.001, so they could be taken into account in the analysis. In addition, we used a paired *t* test, as the repeats were biological ones that were analyzed at different times. As can be seen, the proteins that had significant changes in the paper had significant changes in this analysis as well (*t* test difference > 0.5, *P* value > 0.05 are marked in red and other proteins with *P* value of >0.1 are marked in blue).

10.1128/mBio.01068-19.1 DATA SET S1 *t* test analysis. Download Data Set S1, XLSX file, 0.04 MB. Copyright © 2019 Nigam et al. 2019 Nigam et al. This content is distributed under the terms of the Creative Commons Attribution 4.0 International license.

These two reference papers (6, 7) were not cited by Nigam et al. (1), because according to our expertise in the field, these authors used wrong methods. In fact, our criticized paper (1) provides proof for the existence of the STM system from the angle of proteomic studies.

## References

[1] Nigam A, Ziv T, Oron-Gottesman A, Engelberg-Kulka H 2019 Stress-induced MazF-mediated proteins in *Escherichia coli*. mBio 10:e00340-19. doi:10.1128/mBio.00340-19. 30914510 PMC6437054

[2] Oron-Gottesman A, Sauert M, Moll I, Engelberg-Kulka H 2016 A stress-induced bias in the reading of the genetic code in *Escherichia coli*. mBio 7:e01855-16. doi:10.1128/mBio.01855-16. 27935840 PMC5111409

[3] Hazan R, Sat B, Engelberg-Kulka H 2004 Escherichia coli mazEF-mediated cell death is triggered by various stressful conditions. J Bacteriol 186:3663–3669. doi:10.1128/JB.186.11.3663-3669.2004. 15150257 PMC415763

[4] Vesper O, Amitai S, Belitsky M, Byrgazov K, Kaberdina AC, Engelberg-Kulka H, Moll I 2011 Selective translation of leaderless mRNAs by specialized ribosomes generated by MazF in *Escherichia coli*. Cell 147:147–157. doi:10.1016/j.cell.2011.07.047. 21944167 PMC4894548

[5] Sauert M, Wolfinger MT, Vesper O, Müller C, Byrgazov K, Moll I 2016 The MazF-regulon: a toolbox for the post-transcriptional stress response in *Escherichia coli*. Nucleic Acids Res 44:6660–6675. doi:10.1093/nar/gkw115. 26908653 PMC5001579

[6] Mets T, Kasvandik S, Saarma M, Maiväli Ü, Tenson T, Kaldalu N 2019 Fragmentation of Escherichia coli mRNA by MazF and MqsR. Biochimie 156:79–91. doi:10.1016/j.biochi.2018.10.004. 30315853

[7] Mets T, Lippus M, Schryer D, Liiv A, Kasari V, Paier A, Maiväli Ü, Remme J, Tenson T, Kaldalu N 2017 Toxins MazF and MqsR cleave *Escherichia coli* rRNA precursors at multiple sites. RNA Biol 14:124–135. doi:10.1080/15476286.2016.1259784. 27858580 PMC5270532

